# Affectation in Chronic Invalidism

**Published:** 1874-10

**Authors:** P. H. Hayes


### AFFECTATION IN CHRONIC INVALIDISM.

P. H. HAYES, M. D.

If character cures disease, is want of character among the causes of chronic invalidism? I doubt not I have treated in my life time a score of invalids who could not be cured of their chronic ailments because they did not wish to be cured; they idealised comfortable invalidism. They had long ago discovered that their invalidism made them interesting, brought them consideration and sympathy, furnished an absorbing topic of conversation, their own particular case, and their own remarkable sufferings. Others listen eagerly to these recitals, and for the most part blindly, and often heartily bestow the very expressions of wonder and condolence, which our glad-to-be patient wanted, and which they so well knew how to evoke, and which, when received, confirm them in the habit of watching and nursing, mentally, the things which enter into their false life, and affected heroism.

Is this hard to understand ? and does it sound almost cruel ? Yet there is no moral absurdity in the idea, that the love of sympathy and a romantic desire to be thought heroic in suffering, may become a passion, and be kept up by affectation. And is there not herein a mode of shirking the responsibilities of life, not only without seeming to do it, but with the clear gain of consideration and sympathy ?

If affectation can, through pride or vanity, find its way into dress, manners, language and expression ; if some may, and do, affect wit, learning or eloquence ; nay, if boarding schools teach affectation, and even churches and chapels and prayer meetings are not free from it, then why not other some affect invalidism, if it can only be of a strange kind, and bring the same things, homage, wonder, petting, praise.

As we wish all such invalids might come to character enough to get well at once, we are quite inclined to give some of the symptoms belonging to those who live for the artistic or amateur enjoyment of poor health. They are particularly fond of telling how many skillful doctors—usually from nine to twenty-seven—have tried and failed to cure them, and they tell this with such an air that the new doctor finds the strange impression left upon his mind that his patient seemed pleased in telling of the doctors she had vanquished, and he almost thinks she has a darling property in the strange symptoms which have baffled so many.

In the same breath she begins to tell how many wonderful things the doctors have said about her; that they all agreed they could have cured her if they could only have kept her from over-doing ; that all said she had a peculiarly sensitive, highly-wrought organization; that all agreed they had never seen so strange a combination of diseases before, and never a case that had cost them so much study and compelled such a searching of authorities. Dr. A. said, indeed, there was nothing like it in the books, yet he believed the *liver!* to be the principal seat of the disease, and that there was much neuralgia in the case. Dr. B. said there was congestion of the portal plexus and anemia of the brain. Dr. C. said she had every symptom in the world of consumption of the bowels. Dr. D. gave her thirteen medicines at one time, and some of these medicines she felt sure did not agree with her. Dr. E. declared that her mucus linings were all in rags. Dr. F., Professor in the College, told her that her solar plexus of nerves was in a very adynomic state, and that the reason she could not walk was a want of nerve force in the brain. She thinks Dr. F.

understood her case best of all, had so often felt exactly as he described. She could never forget his devotion! "Poor man, he wore such a long face all the time he was treating me." If she preceives that somehow all this does not impress the new doctor as she had expected, she will very likely add, "Well, doctor, you just wait till I have one of my *spells* and *then* you'll see; you'll have your hands full for once in your life, I guess." The doctor inwardly wonders if she could bring on a "spell" to order. I have known these patients to cry vigorously, declaring they were "frantic with pain," when, as I thought, they were crying from vexation only.

Another, after striving hard to impress me with the number and weight of her maladies, was angry because I would not call them by some high-sounding technical terms, but I said let's call your diseases George Washington and Thomas Jefferson, and that will be large enough, and we can cure you of these names easier than we can of doctor's latin.—Another says to a sister patient, "You are not often depressed, are you? I have always a sad face myself."

Quite characteristic of these patients it is, that whatever other case of strange symptoms may be mentioned, they have had the same symptoms only so much worse. "Dr. B. stood over me and gave me medicine every ten minutes, and Julius left his business and stood over me and fanned me for hours and hours."

Many I have known refuse to eat when other efforts have failed to gain them sympathy and consideration. Others were very fond of considering themselves injured, they could pity themselves and play the character admirably, but when the matter was inquired into, it was too evident that they had sought the opportunity, and enjoyed being a heroic sufferer in the tragedy. One patient would invariably begin to cough as soon as she heard my foot-steps on the stair-case or in the passage.

Now what I have to add is, that the affectation of disease does tend to produce it. Those who, as to bodily symptoms, begin by affecting what they do not feel, may end in feeling in earnest what they had only affected. I knew well a man who as a joke affected stammering, taking off a village character.—After a time he found that when a little excited he could not help stammering, and not to the day of his death could he quite free himself from this habit, only an affectation at the first. A dignified lady affected lisping to divert and amuse her invalid sister, but what was her mortification when in spite of herself she answered the physician, "*Yeth*, doctor." It must not be forgotten that all who affect invalidism and watch symptoms, are in danger of real pains and sufferings. Attention to sensations multiplies and intensifies them. I knew a boy of thirteen who had become so sensitive by having his attention directed to the jerkings of St. Vitus dance, from which he suffered, that he would at once in spite of himself, have any sort of spasm mentioned in his hearing to a third person, and this though the person who spoke of his symptoms was remote from him with his back towards the boy. As soon as his attention was taken off from himself, by never talking of his case in his hearing, and by giving him dishes to carry which he must not break, and liquids he he must not spill, his will was strengthened, his sense of responsibility educated, and a terrible disease finally cured.

Without a doubt many whose invalidism is owing in part to affectation are in a great degree unconscious of it. They have been untruthful and unreal until they are lost. They do not any longer belong to "real folks."

Many of these patients have no real desire to be cured, they go to a new physician to get new cordials. This is the real truth, yet of this I believe they are only dimly if at all conscious. There seems to be an instinctive clinging to that which excuses them from the thought of labor, to that which does not call upon the will for executive force, to that which lets go, in a degree, of the sense of responsibility, and adds on the other side nursing and sympathy.

Many of these patients are the subjects of strong religious emotions, they idealize religion but are not truly religious, they live much in the transcendental cloud-land. But it must be remembered there is no healthy religious feeling apart from religious doing, and all the worked-up states of religious feeling, just for the enjoyment of the thing, are in the nature of affectation, and as unfit to nourish and strengthen the soul as candies and comfits the body.

